# α-Cyclodextrin-Based Polypseudorotaxane Hydrogels

**DOI:** 10.3390/ma13010133

**Published:** 2019-12-28

**Authors:** Adrian Domiński, Tomasz Konieczny, Piotr Kurcok

**Affiliations:** Centre of Polymer and Carbon Materials, Polish Academy of Sciences, 34, M. Curie-Sklodowskiej St., 41-819 Zabrze, Poland; adrian.dominski@cmpw-pan.edu.pl (A.D.); tomasz.konieczny@cmpw-pan.edu.pl (T.K.)

**Keywords:** supramolecular hydrogels, polypseudorotaxane, self-assembly, α-cyclodextrin

## Abstract

Supramolecular hydrogels that are based on inclusion complexes between α-cyclodextrin and (co)polymers have gained significant attention over the last decade. They are formed via dynamic noncovalent bonds, such as host–guest interactions and hydrogen bonds, between various building blocks. In contrast to typical chemical crosslinking (covalent linkages), supramolecular crosslinking is a type of physical interaction that is characterized by great flexibility and it can be used with ease to create a variety of “smart” hydrogels. Supramolecular hydrogels based on the self-assembly of polypseudorotaxanes formed by a polymer chain “guest” and α-cyclodextrin “host” are promising materials for a wide range of applications. α-cyclodextrin-based polypseudorotaxane hydrogels are an attractive platform for engineering novel functional materials due to their excellent biocompatibility, thixotropic nature, and reversible and stimuli-responsiveness properties. The aim of this review is to provide an overview of the current progress in the chemistry and methods of designing and creating α-cyclodextrin-based supramolecular polypseudorotaxane hydrogels. In the described systems, the guests are (co)polymer chains with various architectures or polymeric nanoparticles. The potential applications of such supramolecular hydrogels are also described.

## 1. Introduction

### 1.1. Supramolecular Chemistry

Supramolecular chemistry, as defined by Nobel Prize laureate Jean-Marie Lehn, refers to non-binding interactions that play the same role as covalent bonds in classic organic chemistry. Noncovalent interactions force molecules to associate into highly organized structures, which are characterized by lower bond energy than the typical energy of covalent bonds [1,2]. These supramolecular systems are based on physical interactions, such as van der Waals interactions, hydrogen bonding, π–π dipolar interactions, hydrophobic effects, and host–guest interactions [3,4]. Supramolecular systems are able to freely self-assemble or disassemble because of their noncovalent interactions. These dynamic interactions impart such systems with thixotropic and self-healing properties. This concept can be applied to many novel kinds of supramolecular structures, including catenanes, (pseudo)rotaxanes, and supramolecular hydrogels [5,6,7,8]. Moreover, the application of polymers with desired features (e.g., biocompatibility, biodegradability, stimuli responsivity, defined mechanical, and rheological properties) for the preparation of supramolecular systems is an efficient strategy in biotechnology, chemistry, materials engineering, and biomedicine [9,10,11,12,13,14].

### 1.2. Cyclodextrins

**Figure 1:**
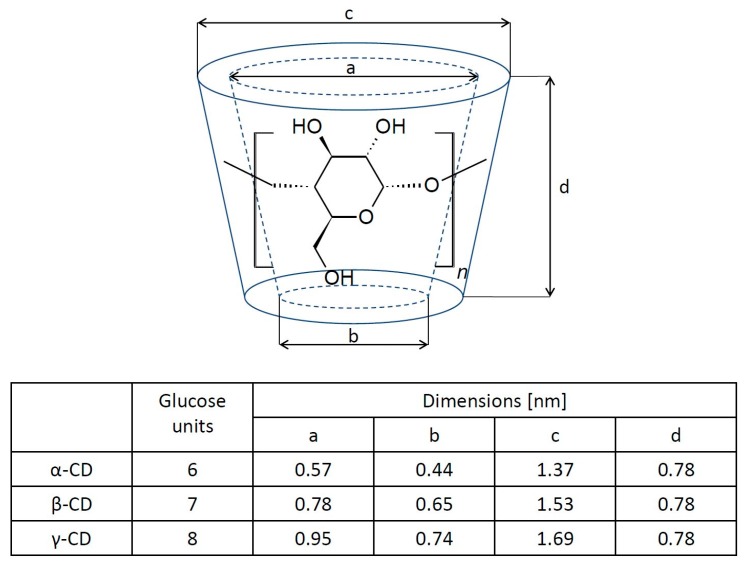
The molecular structure of cyclodextrin molecules and their corresponding geometric dimensions [20].

Cyclodextrins represent a family of macrocyclic oligosaccharides. Generally, cyclodextrins consist of six, seven, or eight glucose units that are linked by α-1-4-glucosidic bonds and they are called α-, β-, or γ-cyclodextrin, respectively [15]. The cyclodextrin (CD) molecule has the shape of a toroid (Figure 1) with a hydrophobic cavity and a hydrophilic outer surface. This is the result of the orientation of the hydroxyl groups in the molecule: the primary hydroxyl groups are in the narrower part, while secondary ones are located in the wider part of the molecule. The cyclodextrin cave is hydrophobic because of the presence of ether-like 1,4-glycosidic linkages [16,17,18]. This structure gives CD one of its most interesting features, namely, the ability to host hydrophobic molecules, which can be incorporated into the hydrophobic cavity [19,20,21]. In an aqueous solution, the outer part of the molecule facilitates interactions with the solvent, while the inner hydrophobic part of the CD molecule favors the formation of an inclusion complex with a hydrophobic “guest” molecule due to solvophobic interactions, such as hydrogen bonding and van der Waals electrostatic interactions [22,23,24,25,26]. The resulting host–guest complex in an aqueous solution is relatively stable. The association constant (K_a_) is defined as:

(1) $$K_{a}=\frac{\left[complex\right]}{\left[guest\right]\left[CD\right]}$$

where [complex], [guest], and [CD] are the concentrations of the components. The association constant, which determines the stability and strength of the complex, is proportional to the quotient of the complex formation constant (k_on_) and its break constant (k_off_) [27,28]. In a water-based solution, the association constant (K_a_) of a guest–host complex that is based on CD can reach 10^5^ M^−1^; for comparison, basic cavitands, such as crown ether, possess K_a_ values that range from 10^2^ to 10^4^ M^−1^ [18,24]. Additionally, cyclodextrins are environmentally friendly and the FDA generally regards them as safe (GRAS) [29]. CDs have a lot of desirable functions, such as catalysis or reversible molecular recognition [17,30,31,32,33]. With this in mind, it is not surprising that they are widely researched and being gradually implemented into the biomedical field [7,26,34].

### 1.3. Supramolecular Cyclodextrin-Based Polypseudorotaxane Hydrogels

Reversible molecular recognition of CDs is one of the most interesting phenomena in supramolecular chemistry. Physical hydrogels that are based on cyclodextrin–polymer inclusion complexes are a family of supramolecular systems and they have gained significant attention over the last 20 years [35,36,37]. Molecular reversible interactions, e.g., ionic/electrostatic or hydrophobic/hydrophilic interactions, hydrogen bonding, crystallization, etc., usually make physically crosslinked hydrogels [7]. Such hydrogels are usually loaded with therapeutic agent during the physical cross-linking process, while the drug can also be loaded in situ in chemically cross-linked hydrogels; however, in this case there is a risk of the drug reaction with cross-linking agent. An alternative to in situ encapsulation might be the incorporation of therapeutic via sorption, however, this is rather inefficient due to the time-consuming and limited loading efficiency. Furthermore, physically crosslinked hydrogels have thixotropic and self-healing properties; therefore, they are easy to use in clinical practice.

There are two main routes for building supramolecular hydrogels: (i) via the formation of inclusion complexes between a cyclodextrin “host” and small “guest” molecule or (ii) by the penetration of particular linear polymeric chains into the cavities of CDs with the formation of “molecular necklaces” that are known as polypseudorotaxanes [37,38,39]. In contrast to a polyrotaxane, in which cavitands are trapped by the use of large molecules (stoppers) at both ends of the polymer chain, cavitands in a polypseudorotaxane (PPR) can be relatively easily de-threaded under appropriate conditions [6]. Harada et al. first reported polypseudorotaxanes [40,41]. The described systems were based on α-CD and high molar mass poly(ethylene oxide) (PEO). The gelation process proceeded by the formation of self-assembled non-soluble crystalline complexes, which acted as physical network knots [42,43]. Since then, supramolecular hydrogels have gained massive attention [44,45,46,47]. However, polypseudorotaxanes, with a few exceptions (β-CD-poly(propylene glycol) and γ-CD-PEG polypseudorotaxane [9,39]) are mainly based on the α-CD with a poly (ethylene glycol) homopolymer or copolymers. Therefore, the aim of this work is to provide a comprehensive understanding of the mechanism, kinetics, and microstructures of polypseudorotaxanes. Further, recent advances in the development of supramolecular hydrogels that are based on host–guest interactions between α-CD and polymers with various architectures are described. Potential applications of the presented supramolecular systems are also discussed. 

## 2. Mechanism and Conditions of Polypseudorotaxane Hydrogel Formation

The conditions of supramolecular hydrogel formation that are based on α-CD–polymer chain polypseudorotaxanes are a basic consideration in the design of novel hydrogels. Most of the systems are based on α-CD and poly(ethylene glycol) (PEG), so further discussion refers to this system. There are at least three different criteria for PPR hydrogel formation. First is the negative binding enthalpy that arises from the local hydrophobic and van der Waals binding interactions between the CD cavity and polymeric chain. Second is the need for a good fit between the CD cavity size and the threading polymer chain. If the guest molecule is too large for the CD cavity, then steric hindrance might result in partial rather than full complexation. On the other hand, if the macromolecule is too small, then it can penetrate into the cavity, but it cannot interact with the CD; consequently, there will be no driving force to create the inclusion complex. The third requirement is that the guest molecule must be soluble or dispersible in an aqueous solution [39,48]. 

**Figure 2:**
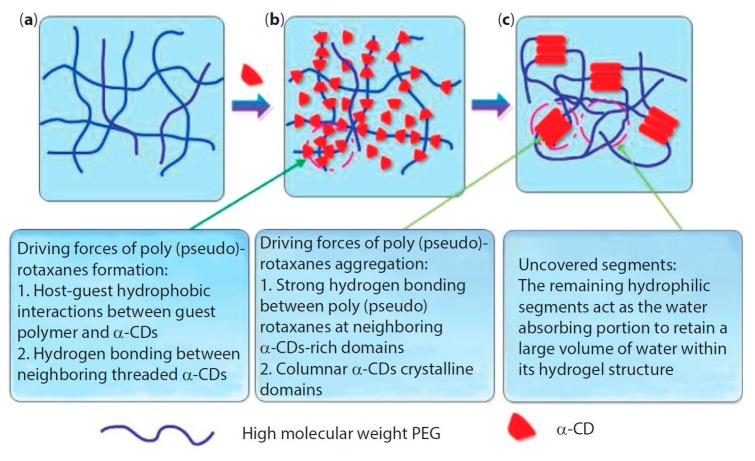
Formation of polypseudorotaxane hydrogels from α-cyclodextrin and high molecular weight poly(ethylene glycol) (PEG). (**a**) PEG aqueous solution without α-cyclodextrin (α-CD); (**b**) α-CD is added and threads onto the polymer chain to form polypseudorotaxanes; and, (**c**) the aggregation of polypseudorotaxanes and exposition of remaining hydrophilic PEG segments give rise to hydrogels. Reprinted with permission from [48]. Copyright (2014) Scrivener Publishing LLC.

The formation of supramolecular PPR hydrogel that is based on α-CD/polymeric chain occurs in two main stages: (i) first, host–guest interactions allow for the polymeric chain to penetrate into the CD cavity with the formation of polypseudorotaxane; (ii) next, hydrogen bonding occurs between neighboring α-cyclodextrin molecules, and these CDs aggregate into crystalline complexes, which act as knots of the physically crosslinked polymer network (Figure 2). However, crystalline complexes rarely form without the appearance of hydrogen bonds between CDs of various PPRs, because of the formation of one-dimensional aggregates of CDs threaded on the polymer chain [49].

As mentioned previously, α-CD possesses two types of hydroxyl groups in a molecular structure: primary hydroxyl groups located in the narrower part (tail) and secondary hydroxyl groups located in the wider part (head). Consequently, CDs can be threaded onto a polymer chain in three different conformations: head-to-head (HH), head-to-tail (HT), and tail-to-tail (TT). Cyclodextrins have been found to mostly arrange in head-to-head and tail-to-tail conformations [50]. However, traces of head-to-tail conformations have also been observed in sequences of CDs that are threaded onto polymer chains [50,51].

**Figure 3:**
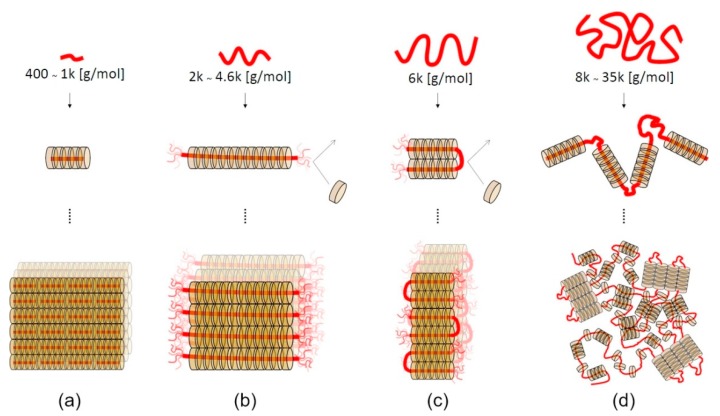
Schematic illustrations of polypseudorotaxane self-assembly mechanisms. (**a**) α-CDs cover the ends of PEG400–1k, and the α-CD crystal grows without limitation of the axis length. (**b**) The ends of PEG2k−4.6k tend to be uncovered by α-CDs because of the entropic gain from the free motion of the axis, which leads to the formation of isolated polypseudorotaxanes with uniform thickness. (**c**) The axis of PEG6k folds to gain chain entropy not only at the ends but also at the folding points. (**d**) For PEG8k–35k, the axis chains fold many times, and remaining uncovered hydrophilic PEG segments give rise to hydrogels. Adapted with permission from *Macromolecules* **2019**, *52*, 3881−3887 [53]. Copyright (2019) American Chemical Society.

The self-assembly behavior of the forming polypseudorotaxanes depends on the length of the PEG chains (Figure 3). For PEG with a relatively small molar mass (400–1000 g/mol), cyclodextrins cover the entire chains, and crystalline domains grow without limitation (Figure 3a) [52]. The ends of PEG chains are not fully threaded by α-CDs when the molar mass of PEG is between 2 and 4.6 kg/mol. Thus, the formation of isolated crystalline domains occurs (Figure 3b). For PEGs with a molar mass of 6 kg/mol, the chains fold into the most favorable conformation, and threaded α-CDs form crystalline domains with uncovered folded PEG chains (Figure 3c). The chains of PEG with a relatively high molar mass (8–35 kg/mol) are folded in many places, and crystalline clusters of α-CD are formed with long uncovered chains (Figure 3d). It should be noted that a small amount of unthreaded α-CD is present in such systems due to the faster kinetics of forming the crystalline domain (gelation) toward the slower threading of the polymer chains [39,53]. The presence of unthreaded PEG chain fragments is necessary for supramolecular hydrogel formation. Hydrophilic PEG chains allow for water to be retained in the structure of the resulting supramolecular polypseudorotaxane hydrogel. Hydrogels cannot be formed without the inclusion of water, because the highly hydrophobic crystalline domains would precipitate in an aqueous solution. The lowest molar mass of PEG that enables α-CD/PEG-based hydrogel formation has been reported to be 2 kg/mol [39].

## 3. α-Cyclodextrin-Based Polypseudorotaxane Hydrogels

Polymers with various molecular architectures may be used in the preparation of α-CD polypseudorotaxanes. As mentioned above, CDs can be threaded onto a linear polymeric chain. However, branched and graft (co)polymers, as well as polymeric nanoparticles, such as micelles and polymersomes, may also be used in the preparation of α-CD/PPR-based hydrogels. Moreover, although most CD/polypseudorotaxane-based supramolecular hydrogels contain poly(ethylene glycol) as the “guest”, since the 1990s, many similar systems have been described based on various polymers, such as poly(propylene oxide) (PPO) [54,55], commercially available Pluronics [56,57,58,59,60,61,62,63,64], and pegylated biodegradable polymers, such as poly(3-hydroxybutyrate) [65,66,67,68], poly(caprolactone) [69,70,71,72,73,74,75,76,77], poly(lactide) [78,79,80], poly(lactide-co-glycolide) [81,82], and polyethylenimine [83,84,85]. Further efforts are focused on the creation of hybrid supramolecular hydrogels with synergistic interactions between CD, polymer, and drug molecules.

### 3.1. α-Cyclodextrin/Linear Polymer-Based Polypseudorotaxane Hydrogels

**Figure 4:**
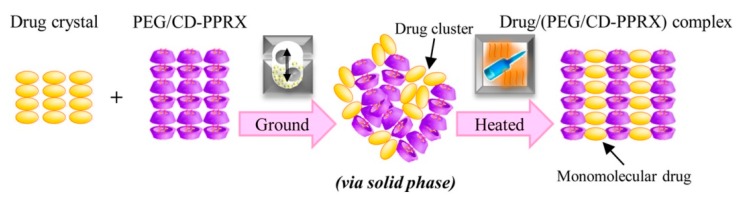
Illustration presenting the formation and structure of a salicylic acid/(PEG/α-CD-polypseudorotaxane) complex. Reprinted with permission from *Cryst. Growth Des.* **2017**, *17*, 1055−1068 [103]. Copyright (2017) American Chemical Society.

The first approach to forming polypseudorotaxanes began with threading α-cyclodextrins onto linear polymeric chains, as described by Harada and Li [40,41]. In these works, hydrogels were the result of forming inclusion complexes between α-CD and high molar mass poly(ethylene oxide) and their subsequent self-assembly in aqueous solutions. In these pioneering works, when the gelation mechanism of the supramolecular hydrogels was investigated, the authors found that poly(ethylene oxide) formed complexes with α-CD in water, and the gels were obtained in a wide range of concentrations. It was found that the gelation time decreased with increasing α-CD and PEG concentrations. It was also revealed that the gelation time increased as the PEG molar mass increased [43]. Further efforts have been primarily directed toward creating polypseudorotaxane hydrogels for biomedical applications, because the components of such hydrogels are biocompatible and nontoxic. Supramolecular hydrogels that are based on α-CD/PEG polypseudorotaxanes are mostly designed as injectable hydrogels for controlled drug release. Li et al. [86] used this approach to create a biocompatible and biodegradable hydrogel as a drug delivery system based on high molar mass poly(ethylene glycol) and α-CD. Hydrophobic drugs were encapsulated at room temperature into the hydrogel matrix in situ without the use of any organic solvents. The erosion of the hydrogel matrix caused the release of the drug. The optimal system was obtained while using PEG with a molar mass that ranged between 20 and 30 kg/mol. Loh et al. modelled the kinetics of biologically active substance release from a similar system based on α-CD/PEG (35 kg/mol) [87]. In this work, the authors studied the effect of the molar mass of the encapsulated drugs on the release kinetics while using two different proteins: bovine serum albumin (BSA; Mw: 67,000 g/mol) and lysozyme (Mw: 14,000 g/mol). The variation in protein concentration did not affect the rate of drug release. The authors observed that the largest effect on the kinetics of drug release was the size of the vials in which the experiment was conducted. The release profile was prolonged in vials with a smaller diameter, and the amount of released substance was reduced. The linear relationship between the hydrogel mass loss and the protein release profile confirmed that the erosion of the hydrogel matrix is the key factor for drug release. Such systems that are based on α-cyclodextrin and poly(ethylene glycol) have found wide applications as a drug carriers for low molar mass drugs; nucleic acids; or proteins, such as insulin [88,89], lysozyme [90], human immunoglobulin G [91], antibody-based drugs (such as omalizumab, palivizumab, panitumumab, and ranibizumab [92]), α-chymotrypsin [93], Jack bean urease [94], silver sulfadiazine [95], and siRNA [96]. Recently, systems with nucleobase-terminated PEG were synthesized to improve the rheological properties and prolong drug release. Nucleobase (adenine/thymine)-terminated poly(ethylene glycol)s obtained injectable, biodegradable, and biocompatible supramolecular hydrogels. The base-pairing interactions between adenine and thymine, as occurs in the structure of the DNA double helix, enhanced the mechanical properties of the hydrogel. Additionally, in vivo experiments confirmed that a doxorubicin (DOX)-loaded hydrogel exhibited very good biocompatibility, an appropriate drug release profile, and inhibitory effects on tumor growth that were much more effective than free doxorubicin [97]. Mixing nucleobase (guanine/cytosine)-terminated poly(ethylene glycol)s with α-cyclodextrin was undertaken to develop a similar system. The obtained supramolecular hydrogel was thermo-responsive and also showed enhanced mechanical properties due to an additional network junction that formed by base-pairing [98]. Such a simple and convenient anticancer drug delivery system could be used as a thermo-controlled drug delivery system because tumoral temperature is higher than that of normal tissues as a result of the increased metabolic rate of tumor cells [99]. A similar system with chemically modified poly(ethylene glycol)s was developed by Wa et al. [100]. Ferrocene functionalized the low molecular weight poly(ethylene glycol) as a mono end-group. Ferrocene forms inclusion complexes with α-CD by itself. The synthesized α-ferrocene–PEG with α-CD created a supramolecular hydrogel, which showed shear-thinning properties due to dual host–guest interactions between CD and ferrocene–PEG. Other desired properties can be obtained by subjecting cyclodextrin to chemical modifications, such as grafting with appropriate PEG chains to achieve thermal behavior. Recently, various polymers that are grafted with PEG chains have attracted massive interest for their thermal behavior induced by PEG side chains [101]. While considering this behavior, Arima et al. [102] prepared thermo-responsive hydrogels from an α-cyclodextrin derivative (2,6-di-O-methyl-cyclodextrin) and polyethylene glycol or polypropylene glycol in water at temperatures of >50 or >35 °C, respectively, but they did not form at room temperature. Ogawa et al. [103] suggested that drugs could be incorporated into the intermolecular spaces of cyclodextrin crystalline columns in α-CD/PEG polypseudorotaxanes. The guest drug (salicylic acid) with α-CD from polypseudorotaxanes was first amorphized (by co-grinding), and then the crystallization of CDs caused by subsequent heating resulted in the formation of a salicylic acid/(PEG/α-CD-polypseudorotaxane) complex, in which the drug is incorporated into cyclodextrin crystalline columns, as a result of specific solid-phase-mediated co-grinding with subsequent heating (Figure 4). 

**Figure 5:**
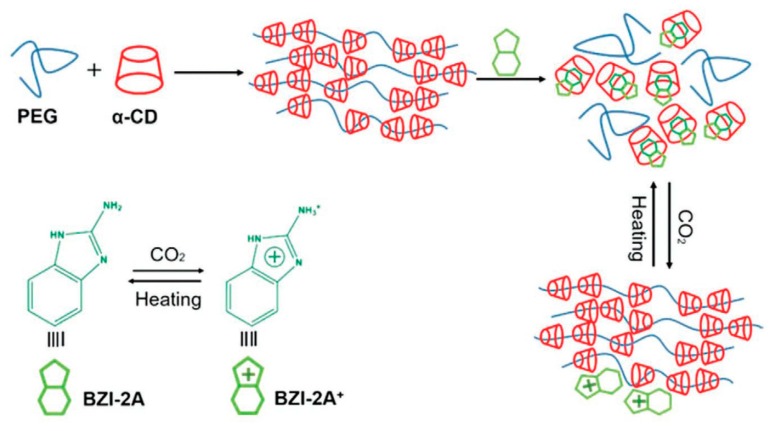
Preparation of the responsive polypseudorotaxane hydrogels triggered by CO_2_. Adapted with permission from *Macromol. Chem. Phys.* **2019**, *220*, 1900071 [112]. Copyright (2019) WILEY-VCH Verlag GmbH & Co. KGaA, Weinheim.

Some challenges remain in making these hydrogels attractive for biomedical applications, although most α-CD-based polypseudorotaxane supramolecular hydrogels are still based on poly(ethylene glycol) chains. For example, the use of high molecular weight PEG to obtain stable hydrogels is a problem, because human kidneys cannot filter out such large polymer molecules since their hydrodynamic radius is very large [9]. Therefore, a number of new systems have been developed to make these supramolecular hydrogels more practical for a variety of applications in the biomedical field. As α-cyclodextrins are capable of “sliding” along some polymer chains, similar to beads on a necklace, they are often aptly named “sliding gels”. This ability of α-cyclodextrins to slide on some polymer chains and interact with other ones makes the sequence of the block copolymer chain extremely important. Moreover, the possibility of easily manipulating the chain sequence while using a massive number of novel controlled polymerization methods (e.g., ATRP, RAFT) has enabled the threading of α-cyclodextrins in the desired place on the block polymer chain. This process allows for many possibilities to obtain hydrogels that leave an “intelligent” functional group free while threading cyclodextrins on the inactive part of the polymer chain to obtain supramolecular hydrogels. A triblock copolymer can be designed with the desired block sequence for the preferential threading of α-CDs, such as in commercially available Pluronics (PEG-PPO-PEG) ABA triblock copolymer: α-CD molecules can only thread along the “A” blocks for the formation of supramolecular hydrogels [56,57,58]. Such systems have been studied for controlled drug delivery. Li et al. [59] showed that the Pluronics/α-CD-based polypseudorotaxane system has thixotropic properties, which can be used for protein delivery systems. The copolymer composition has a significant impact on the release profile of the active substance. It turned out that the optimal system contained 30 mol% of poly(ethylene glycol). Systems that are based on well-known commercially available Pluronics, such as P123 and P127, have also been developed and used for the release of biologically active substances, such as antibiotics (Vancomycin) [60] and antifungal medications (Griseofulvin) [61], or gene delivery [62]. A similar approach might be used to create a PPR, wherein α-CD forms an inclusion complex only with the middle “B” block in an ABA copolymer. For example, α-CD is not able to thread on PPO blocks and form an inclusion complex in commercially available Pluronic R series (PPO-PEO-PPO). However, α-CD can slide through PPO chains, and it selectively threads on the middle PEO block with complex formation because the enthalpy of complex formation is higher than the energy resistance of α-CDs penetrating through the PPO chains [63]. Furthermore, Laranetta et al. [64], while studying the mechanism of hydrogel formation with ABA copolymers of PPO-PEO-PPO, where α-CDs can only be threaded on the middle “B” block of the copolymer, stated that the hydrophobic interactions of the outer PPO blocks are mainly responsible for the formation of hydrogels. Polypseudorotaxanes that were based on an ABA copolymer with poly(3-hydroxybutyrate) (PHB) as the middle block were also prepared. Synthetic and natural poly(3-hydroxybutyrate)s are very popular in biomedical applications due to their excellent biodegradability and biocompatibility. PHB can be synthesized by biotechnological processes or as a result of a chemical reaction, such as the polymerization of β-butyrolactone [104]. The mixing of the amphiphilic PEG-PHB-PEG triblock copolymer with α-CD synthesized a hydrogel. Both polypseudorotaxane hydrogels showed increased stability, regardless of whether bacterial crystalline isotactic poly([R]-3-hydroxybutyrate) [65,66,67] or synthetic atactic poly([R,S]-3-hydroxybutyrate) [68]. The increase in stability is due to the hydrophobic interactions between PHB blocks, which might be useful for prolonged controlled drug release. This same approach can be used to produce hydrogels with poly(ε-caprolactone) (PCL), which is a biodegradable polyester that is widely used in biomedical applications. Ring-opening polymerization of ε-caprolactone using anionic, cationic, and coordination catalysts or via free-radical ring-opening polymerization of 2-methylene-1-3-dioxepane can synthesize PCL [105]. Biodegradable amphiphilic hydrogels based on the poly[(ethylene oxide)-b-ε-caprolactone] (PEO-b-PCL) AB diblock copolymer [69], wherein α-CD can also be threaded onto the poly(ε-caprolactone) “B” block, were obtained. However, in such a system, the amphiphilic copolymer prefers to aggregate into micelles with a PCL core because of the strong hydrophobicity of PCL. This configuration prevents the threading of α-CDs onto PCL chains located in the micelle core. As poly(ε-caprolactone) has excellent biodegradability, such hydrogels have been prepared for controlled drug release. For instance, it was successfully used for the release of dextran labeled with fluorescein [69], doxorubicin (DOX), and DOX/cisplatin [70]. However, triblock ABA and BAB copolymers consisting of PCL and PEG blocks have also been developed for drug release because of the ability of α-CD to thread onto PCL chains. Tang et al. [71] investigated whether local intramyocardial injection of the α-CD/PEG-PCL-PEG polypseudorotaxane hydrogel with encapsulated erythropoietin could increase cardioprotective effects without causing polycythemia after myocardial infarction. In vivo experiments showed the sustained release of erythropoietin, which reduced the infarct size and improved cardiac function, increased neovasculature formation, and inhibited cell apoptosis. These systems have a prolonged drug release profile because of the slow in vivo enzymatic degradation of PCL [106]. For example, in vivo tests of hydrogels prepared from PEO-PCL-PEO and α-CD have shown total biocompatibility, which makes them promising candidates as injectable scaffolds for tissue engineering applications [72,73], and they enabled insulin to be released for over 18 days, which is a promising result for a release system of insulin [74]. Similar properties were obtained for supramolecular hydrogels that were prepared from PCL-PEO-PCL. This system also has excellent biocompatibility and rheological properties that can be used as tissue-engineered scaffolds [75], with the sustained release of vitamin B12 or naltrexone [76] and insulin [77] over a period of 20 days. Recently, some efforts have focused on the creation of composite hybrid PPR hydrogels by including carbon materials instead of modifying the main chain. For example, the addition of graphite nanoplates accelerates the speed of gel formation, improves water retention ability, and leads to better sustained release for the hybridized supramolecular hydrogels when compared with the native hydrogels [107]. Other carbon materials have been successfully used to prepare hybrid polypseudorotaxane hydrogels. Wang et al. [108] prepared a triple-responsive graphene oxide hybrid hydrogel. Graphene oxide sheets acted as the core material, providing additional crosslinking, and they also absorbed near-infrared (NIR) light and converted it into heat to trigger a gel−sol transition. Similar graphene oxide hybrid supramolecular hydrogels were used for controlled drug delivery. The graphene oxide hybrid supramolecular hydrogel efficiently loaded two anticancer drugs at various proportions. The release of the drugs in an on-demand and dual-phase fashion upon stimulation by NIR light enabled better dynamic control of the drugs than the traditional diffusion-based mode [109]. Xin et al. used the same types of carbon materials [110] for the modification of a hydrogel based on α-CD and the nonionic liquid polymer Tyloxapol. This hydrogel was formaldehyde responsive. These hydrogels can be used as formaldehyde sensors and/or for the removal of formaldehyde from home furnishing, which might be beneficial for reducing indoor pollutants, because a small amount of formaldehyde induces a gel-to-sol transition. At the same time, the addition of graphene or graphene oxide to α-CD/PEG supramolecular hydrogel significantly changed their microstructure and rheological properties. Moreover, it also induced fluorescence quenching, and the resulting hydrogel could be used as an optical sensor [111]. Small molecules can also be used to trigger phase transitions of supramolecular hydrogels. For example, the addition of 2-aminobenzimidazole induced CO_2_ responsiveness. Adding 2-aminobenzimidazole changed the obtained PEG/α-CD hydrogels into a solution, which can replace PEG chains because 2-aminobenzimidazole with α-CD has a higher association constant (K_a_) than PEG. Purging the solution with CO_2_ regenerates the supramolecular hydrogel (Figure 5) [112].

### 3.2. α-Cyclodextrin/Branched Polymer-Based Polypseudorotaxane Hydrogels

Besides the linear “guest” polymer chains, other branched polymers have also been used for PPR preparation. It was first described by Goh et al. [113], who prepared polypseudorotaxane hydrogels that were based on star-shaped PEG with three, four, and six arms. Such star PEGs form inclusion complexes with α- and γ-CD. In their pioneering work, Harada and Li [114] stated that two chains of poly(ethylene glycol) could be simultaneously threaded through the γ-CD cavity. Similarly, the supramolecular polypseudorotaxanes were prepared by the inclusion complexation of high molecular weight star-poly(ethylene glycol) containing 13 and 15 arms with α- and γ-CD [115]. The use of this approach resulted in some promising supramolecular hydrogels that could be applied as controlled drug delivery systems. Yang et al. [116] developed a polypseudorotaxane hydrogel made of four-arm PEG and α-CD. The hydrogel had thixotropic properties and it underwent a reversible gel–sol phase transition in response to shear stress changes. This simple hydrogel was highly biocompatible, and shear stress controlled the kinetics of drug (brimonidine) release. Sedlak et al. [117] developed pH-triggered supramolecular hydrogel that was based on an α-CD/4-arm PEG conjugate with prednisolone as an alternative application for the peroral administration of drugs. After 1 h in a model stomach environment, 86% of conjugated prednisolone from the initial polypseudorotaxane hydrogel remained unchanged and could be transported further through the digestive tract. A similar supramolecular hydrogel that was based on a hyperbranched polyglycerol, poly(ε-caprolactone), and poly(ethylene glycol) was successfully used for sustained anticancer drug release. In vivo studies of this hydrogel showed blood compatibility and non-cytotoxicity, which make this system a good candidate for application in tumor therapy [118]. Polypseudorotaxane hydrogels that were prepared from multi-arm β-CD-6-poly(ε-caprolactone) (with an average of six PCL chains that were selectively connected to the wider side of β-CD) with a unique “jellyfish-like” structure and α-CD are also promising for application in the biomedical field [119]. Currently, studies are focused on improving the rheological properties of hydrogels that are based on branched polymers to give them stimuli-responsive properties and find new applications for them. For example, Isasi et al. used the polypseudorotaxane hydrogel based on commercially available four-armed poloxamines (Tetronics^®^) for sustained protein delivery [120]. The gel is easily eroded, so the controlled release of encapsulated large molecules, such as proteins, is possible, and it can be tuned by varying the α-CD/copolymer ratio. On the other hand, the same system has been used for green chemical catalyst-containing media [121,122]. For instance, α-CD/Tetronics composition was applied for the rhodium-catalyzed hydroformylation of alkenes [123]. Moreover, gold nanoparticles that were embedded in the Tetronic/α-CD hydrogel matrix were used as catalysts for the hydrogenation of alkenes, alkynes, and aldehydes [124]. Another approach is based on simply mixing carbon-based materials with the obtained supramolecular hydrogels. Introducing graphene or graphene oxide into supramolecular hydrogel based on a α-CD and seven-arm star-like branched PEO-PPO-PEO block copolymer significantly improved the dye adsorption capacities. At the same time, incorporating graphene and graphene oxide successfully modulate the morphology and the mechanical strength of the native hydrogel [125]. Similarly, the addition of graphene oxide to PPR hydrogels prepared from α-CD and commercially available poloxamine (reverse Tetronic 90R4, T90R4), which has four diblock arms with a PPO–PEO structure, produces a hydrogel that can selectively adsorb various dyes, while the native one cannot adsorb the dyes at all. The native hydrogel has been used for controlled drug release because it has good biocompatibility. Moreover, the addition of graphene oxide to this system prolongs the drug release profile [126]. 

In addition to star-like copolymers, polypseudorotaxane hydrogels that are based on dendrimers can be prepared. These supramolecular constructs have mostly been prepared for controlled gene delivery. The erosion of supramolecular hydrogel leads to prolonged release of genes in the form of a polyplexes with increased transfection efficiency compared to the free gene. Tuning the CD concentration to obtain sustained gene release can control the degree of hydrogel matrix erosion. The transfection efficiency can be adjusted by the application of respective cationic (co)polymer and its molar mass. Polyplexes with constant transfection efficiency can be released in a sustained manner for several days [127]. Zhang et al., who prepared a pegylated polyamidoamine dendron that was used to condense plasmid DNA into a polyplex, first demonstrated the concept of dendrimer-based supramolecular hydrogels [128]. Subsequently, it was encapsulated by the supramolecular hydrogel with α-CD and exhibited a sustained release profile and active gene transfection properties. Recently, Xue et al. [129] prepared a supramolecular hydrogel while using α-CD and a pegylated arginine-functionalized poly(l-lysine) dendron for MMP-9 shRNA plasmid delivery. In vivo studies have shown that the obtained pMMP-9-loaded supramolecular hydrogel possesses good biocompatibility and sustained tumor growth inhibition. Arima et al. [130,131] developed a series of biodegradable polypseudorotaxane systems that were based on pegylated polyamidoamine dendrimers conjugated with α-cyclodextrin and α- and γ-CD for novel sustained DNA release. For example, a polypseudorotaxane of a poly(ethylene glycol)-grafted α-CD/polyamidoamine dendrimer conjugate was used as a sustained release system for plasmid DNA. In vitro and in vivo studies revealed that the hydrogel enabled the sustained release of plasmid DNA for at least three days. Additionally, this system showed sustained transfection efficiency for 14 days after its intramuscular injection into mice. These results indicate that these supramolecular hydrogels are a fully biocompatible and, thus, a promising candidate for sustained DNA release systems. 

### 3.3. α-Cyclodextrin/Grafted Polymer-Based Polypseudorotaxane Hydrogels

**Figure 6:**
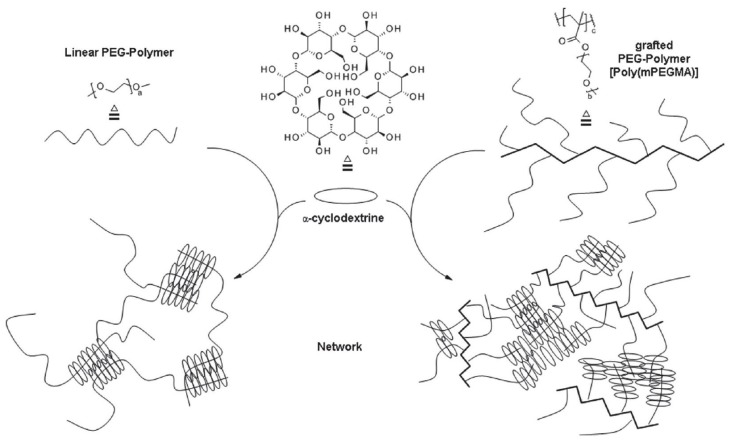
Schematic structure of PPR hydrogels made from linear (left) and PEG-grafted copolymer (right) with α-cyclodextrin. Adapted with permission from *Macromol. Chem. Phys*. **2019**, *220*, 1900081 [132]. Copyright (2019) WILEY-VCH Verlag GmbH & Co. KGaA, Weinheim.

Graft copolymers have a unique architecture when compared with linear or star-shaped copolymers. The presence of side chains at every repeating unit leads to a higher degree of crosslinking (Figure 6), and polypseudorotaxane hydrogels with better rheological properties are obtained. 

Yui et al. [133] described a supramolecular hydrogel that was synthesized while using PEG-grafted dextrans and α-CD in one of the earliest reports concerning PPR based on PEG-grafted copolymers. α-CD assembly and disassembly induced the gel–sol transition, and the transition was reversible with hysteresis. Varying the polymer concentration and the PEG content in the side chains of copolymers, as well as the stoichiometric ratio between the α-cyclodextrin and grafted PEG chains, controlled the transition temperature. Improving the rheological properties of such an excellent biocompatible and biodegradable hydrogel would make this dextran-based supramolecular hydrogel useful in the biomedical field for applications, such as an injectable drug delivery system or tissue engineering scaffold. Similarly, Yui et al. [134] demonstrated a pH- and thermo-responsive polypseudorotaxane hydrogel while using a poly(ε-lysine)-grafted dextran and α-CD. The obtained hydrogel showed reversible gel–sol phase transitions upon heating and cooling. Moreover, introducing cationic side chains induced pH sensitivity. In an acidic environment, protonation on the grafted poly(ε-lysine) chains caused a rapid de-threading of α-cyclodextrin molecules. Thus, the reversible decomposition or formation of polypseudorotaxane hydrogel, depending on the medium pH, was observed. Similarly, relatively high molar mass PEG-grafted biodegradable polypseudorotaxanes have been prepared. PEG chains were grafted onto chitosan [135], hyaluronic acid [136], glucomannan [137], alginate [138], and heparin [139], and various factors affected the gelation properties of the resulting materials, including the substrate ratio and concentration, temperature, pH, and grafted PEG content. Very recently, Hartwig et al. [132] studied the kinetics of supramolecular hydrogel formation and the mechanical properties resulting from structural changes of the polymer. For this study, a PPR/α-CD hydrogel that was based on acidic copolymers with PEG side chains made of methoxy-PEG methacrylate and methacrylic acid (MA) was prepared. The gel formation was pH-dependent due to the methacrylic acid units present in the copolymers. The results revealed that the incorporation of MA groups as grafted chains increased the hydrogel strength and density. Wang et al. [140] reported research on the pH- and temperature-responsive sol–gel transition of a supramolecular hydrogel. Being prepared via the ATRP technique, PEG-grafted copolymers with protonatable segments and poly[poly(ethylene glycol) methyl ether methacrylate]-co-poly[2-(dimethylamino)ethyl methacrylate] formed polypseudorotaxane hydrogels with α-cyclodextrin. The authors showed that the density of PEG branches and the chain uniformity of the copolymer affected the gelation behavior. It was also found that temperature and pH controlled the protein release kinetics. Loh et al. [141] prepared a hydrogel based on biodegradable pectin-graft-methoxy-poly(ethylene glycol) methacrylate copolymer synthesized via radical polymerization initiated by cerium(IV). These supramolecular hydrogels possessed thixotropic properties. However, the α-CD/pectin-PEGMA hydrogels were not affected by temperature in the range of 10–40 °C in contrast to most of the other polypseudorotaxane hydrogels. It was hypothesized that pectin stabilizes these hydrogels in the investigated temperature range. Biodegradable PEG-grafted polymers were also prepared while using poly(organophosphazenes) as the main chain [142]. The obtained results showed that the gelation time depended on the molar mass of PEG side chains, the molar ratio between PEG repeating units and α-cyclodextrin content, and the concentration of the polymeric gel precursors. This hydrogel has potential as an injectable drug delivery system because of its excellent biocompatibility and biodegradability, which was tested by using bovine serum albumin (BSA) as a model compound. Hydrogels with longer PEG side chains showed better stability and prolonged BSA release. Similarly, Xin et al. recently described a promising candidate as an injectable drug delivery system [143]. Hydrogels were prepared using α-CD and various silicone surfactants. The results indicated that the balance between the hydrophilicity and the hydrophobicity of the silicone surfactants is crucial in the formation and stability of the hydrogels. A silicone polymer that was obtained using monomers with two PEG substituents exhibited a stronger ability to form hydrogels when compared with silicone possessing only one PEG chain in the repeating unit, regardless of the molar mass of PEG. Moreover, an in vitro study using doxorubicin showed that a α-CD/silicone surfactant polypseudorotaxane hydrogel could be used as an injectable carrier for controlled drug release. Recently, Schmidt et al. [144,145] synthesized a thermo-adaptive supramolecular hydrogel that was based on α-CD and a double hydrophilic block copolymer (DHBC), [poly(N-vinylpyrrolidone)-b-poly(oligo ethylene glycol methacrylate)]. Heating the DHBC-based hydrogel above T_sp_ (sol point) and then cooling it to ambient temperature led to weak-flowing gels. On the contrary, heating above T_cp_ (cloud point) and cooling to an ambient temperature led to a hydrogel with properties that were similar to the initial hydrogel. The mechanical properties of the supramolecular hydrogel depend on the thermal history of the material, which is probably because of the DHBC-mediated modulation of crystallization, which can be an interesting material for applications in sensing. Similarly, a DHBC copolymer was used to prepare a pseudorotaxane hydrogel from a comblike PEO-grafted triblock polymer (P2VP-b-PPEOMA-b-P2VP) that was synthesized via the RAFT technique with α-cyclodextrin. However, the results showed that the obtained polypseudorotaxane was water-soluble as a result of the presence of poly(2-vinylpyridine) (P2VP) blocks that were long enough to prevent the precipitation of PPR when adding a small amount of α-CD (26 mg/mL). When a greater amount of CD (84 mg/mL) was added, hydrogel formation was observed. The authors stated that the water solubility of the polymer might affect, to a certain extent, the number of inclusion complexes formed [146]. Yang et al. [147] described an interesting strategy for the in situ formation of polypseudorotaxane hydrogels through the photoinitiated copolymerization of poly(ethylene glycol) methyl ether methacrylate, acrylamide, sodium acrylate, and α-CD in a water-based solution.

### 3.4. α-Cyclodextrin/Polymer Brush-Based Polypseudorotaxane Hydrogels

**Figure 7:**
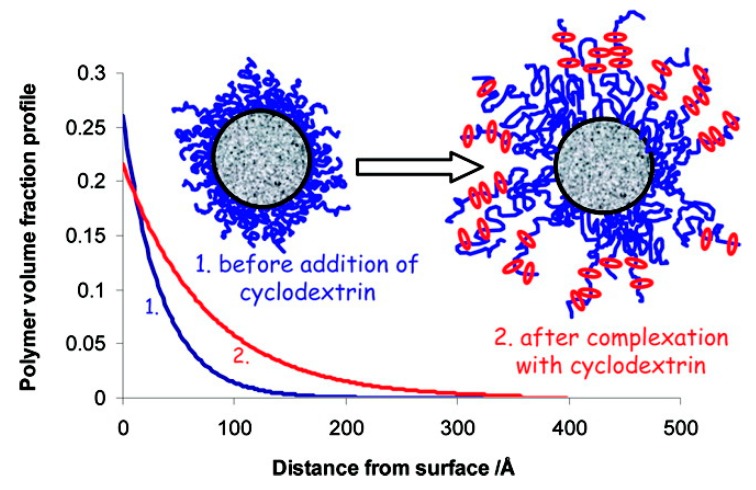
Plot presenting the effect of adding α-CD on the polymer volume profile. Adapted with permission from *Langmuir* **2008**, *24*, 10005–10010 [149]. Copyright (2008) American Chemical Society.

Similarly, high-density brush polymer architecture was also explored for polypseudorotaxane hydrogel formation. In the very first report, Liu et al. [148] described the preparation of hydrogels that were based on a series of double-grafted polymer brushes, PBIEM-graft-P(PEOMA), being synthesized using the “grafting from” approach via ATRP of poly(ethylene glycol) methyl ether methacrylate (PEOMA) while using a well-defined macroinitiator, poly(2-(2-bromoisobutyryloxy)ethyl methacrylate) (PBIEM). Later, Dreiss et al. [149] studied the interaction between PEG layers that were grafted on colloidal particles. It was found that the addition of α-CD affected the structure of the grafted layer, which caused the extension of PEG chains into the bulk, allowing for the formation of a polypseudorotaxane (Figure 7). Recently, Yokoyama et al. [150], investigated the formation of inclusion complexes between α-CD and a poly(ethylene glycol) brush and observed a similar phenomenon. It was found that a polypseudorotaxane consisting of randomly oriented α-CD polycrystals appeared when the PEG brush was exposed to a 10% α-CD solution. However, when the PEG brush was exposed to a 5% α-CD solution, a uniform 10 nm thick polypseudorotaxane layer with α-CD stacked perpendicular to the substrate was formed.

The use of a polypseudorotaxane hydrogel that was based on brush-like polymers was also explored for biomedical applications. Brush-like polyethylene glycol methyl ether methacrylate copolymerized the multi-armed polyglycerol sebacate cores via the ATRP technique. This biodegradable and biocompatible hydrogel possessed a tunable low upper critical solution temperature (<90 °C) and self-healing ability, and it was comparable to a human soft tissue modulus (around 100 kPa). Such hydrogels can find potential applications in tissue engineering, cosmetics, and skincare [151].

### 3.5. α-Cyclodextrin/Nanovehicle-Based Polypseudorotaxane Hydrogels

The rapid progress in biomaterials and nanotechnology has allowed for the precise design of hybrid and multifunctional colloidal particles that are different in type, size, and shape. Hence, their possible applications as stimuli-responsive or target-oriented carriers are enhanced. The assembly of single macromolecules into high-order structures, such as micelles, capsules (incl. vesicles), nanoparticles, nanofibers, and hydrogels, are currently of interest to many scientists. It has become a well-controlled process, because a single component of a highly ordered structure can be easily tailored by various parameters, such as the molar mass and hydrophilicity/hydrophobicity ratio of the copolymers used and the addition of smart stimuli-responsive groups for pH, thermal, and light sensitivity, which can be used to trigger a reversible conformation from a high-order morphology to a lower ordered structure [152]. The addition of α-CD to these nanovehicles can result in a change in the overall properties. By taking advantage of supramolecular chemistry, Loh et al. [153] studied a system of α-CD/PEG-containing micelles to provide a basis for understanding the interactions between micelles and α-CD in PPR hydrogel formation. The supramolecular hydrogel architecture was obtained in two steps: (i) micelle formation as a result of the self-assembly of the amphiphilic poly(PLLA-DMAEMA-PEGMA) copolymer in an aqueous solution; and, (ii) micelle association with α-CD from the threading of PEGMA chains in the α-CD cavity to form inclusion complexes that act as polymer network knots.

**Figure 8:**
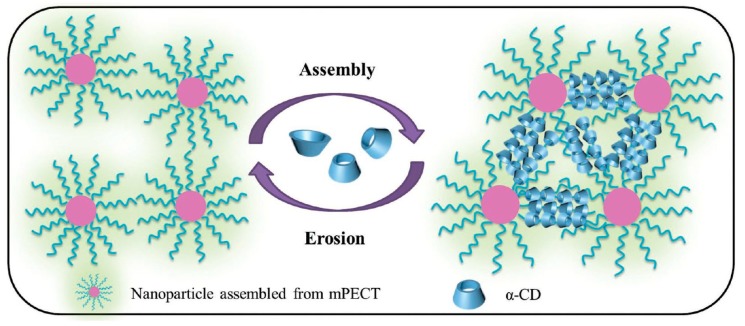
Illustration of the formation and structure of a polypseudorotaxane hydrogel composed of nanoparticles and α-CD. Adapted with permission from *Macromol. Biosci*. **2016**, *16*, 1188−1199 [154]. Copyright (2016) WILEY-VCH Verlag GmbH & Co. KGaA, Weinheim.

Deng et al. [154] outlined the usefulness of injectable polypseudorotaxane hydrogels that were prepared from biodegradable micelles in biomedical applications (Figure 8). While using the amphiphilic block polymer poly(ethylene glycol)-b-poly(ε-caprolactone-co-1,4,8-trioxa[4.6]spiro-9-undecanone), the authors showed that the release behavior could be modulated by changing the concentration of the nanoparticles or α-CD. Furthermore, extended in vivo cytotoxicity studies demonstrated its biodegradability and biocompatibility. The supramolecular hydrogel that formed after in vivo subcutaneous injection showed a retention time of approximately 14 days. PPR hydrogels’ remarkable features strongly indicate that such hydrogels have potential clinical applications for local drug-loaded nanoparticle delivery. Recently, Yang et al. [155] prepared biocompatible and biodegradable polypseudorotaxane hydrogel by the host–guest interaction between α-CD and PEG chains of poly(ethylene glycol)-b-poly(lactic acid) micelles. The synthesized hydrogel had shear-thinning and self-healing properties. Further, doxorubicin was encapsulated into the micelles. Release from the hydrogel matrix was sustainable, with the release rate dependent on the α-CD concentration. The released DOX showed higher inhibition efficacy against HeLa cells when compared with free DOX. A simple biodegradable and biocompatible system based solely on PEG–PLA micelles and α-CD has potential as an injectable system for in vivo drug delivery. Similarly, a biodegradable and versatile injectable supramolecular hydrogel that was based on α-CD/micelles prepared from glycol chitosan–Pluronic F127 conjugate, as reported by Yao et al. [156], was used to encapsulate anticancer drugs: hydrophobic doxorubicin or hydrophilic 5-fluorouracil, as well as the protein superoxide dismutase. The α-CD concentration tuned the mechanical properties and dissociation rate of the PPR hydrogel. This hydrogel showed a prolonged and almost linear release profile for various biologically active substances that were loaded. The hydrogels released free drugs as well as drug-loaded micelles via the erosion of the hydrogel matrix under physiological conditions. An injectable micellar supramolecular hydrogel that was composed of PEG_5000_-b-PCL_5000_ micelles and α-CD was successfully prepared and used for the encapsulation of paclitaxel (PTX). This system has a tunable gelation time, mechanical thixotropic properties, and release profile of drugs from the hydrogel. Furthermore, this biodegradable hydrogel increased the biological activity of encapsulated PTX when compared with free PTX, as was indicated by an in vitro cytotoxicity assay [157]. Li et al. used the same PEG-b-PCL micelles [158] to prepare a hydrogel with α-CD for topical ocular drug delivery. This system exhibited thixotropic properties, which are beneficial to ocular drug delivery. In vivo studies indicated that the hydrogel had low cytotoxicity and a sustained drug (diclofenac) release profile, and it was a nonirritant toward the rabbit eye and it could significantly improve ocular drug bioavailability. These properties are highly desirable, because, in typical eye drops that are currently used, only less than 1% of the applied drug is effective, since tear turnover, drainage, and lacrimation immediately eliminate the majority of the drug [159]. Recently, Hirayama et al. [160] developed a temperature-dependent reversible sol−gel transition PPR hydrogel made of hydroxypropyl methylcellulose and α-CD for an ocular drug delivery system. The potency of this system in an ophthalmic formulation was tested on rabbit eyes. This supramolecular hydrogel possessed low viscosity at room temperature, which made it easy to administer, but it rapidly formed a viscous hydrogel on the ocular surface at physiological temperature, which improved the ocular absorption of the drug (diclofenac). Very recently, the formation of polypseudorotaxane hydrogels while using a mixture of two types of micelles for the ocular delivery system of natamycin was developed. Natamycin was encapsulated by Soluplus (polyvinyl caprolactam–polyvinyl acetate–polyethylene glycol graft copolymer) amphiphilic copolymer and Pluronic P103 micelles as well as α-CD. The application of this mixed micelle system showed different natamycin diffusion coefficients and permeability values of the Soluplus/Pluronic-based hydrogel. Therefore, the preparation of a mixed micelle system might be a useful tool for regulating drug release and increasing ocular permeability [161]. However, the possibility of easily tuning the rheological behavior and drug release pattern by changing the ratio and concentration of copolymer and α-CD enables the use of such hydrogels for desired applications. For example, Soluplus micelles/α-CD PPR hydrogel was recently developed for the transdermal drug delivery of carvedilol [162]. Li et al. [163] described a new glucose-responsive, three-component hydrogel (poly(ethylene glycol)-b-vinyl alcohol), α-cyclodextrin, and phenylboronic acid (PBA)-terminated PEG) for insulin delivery. Poly(ethylene glycol)-b-poly(2-(diethylamino)ethyl methacrylate) micelles/α-CD supramolecular hydrogel was proposed for carrying radiotherapeutic metal ions Y(III) and Cu(II) for internal tumor imaging and therapy [164].

**Figure 9:**
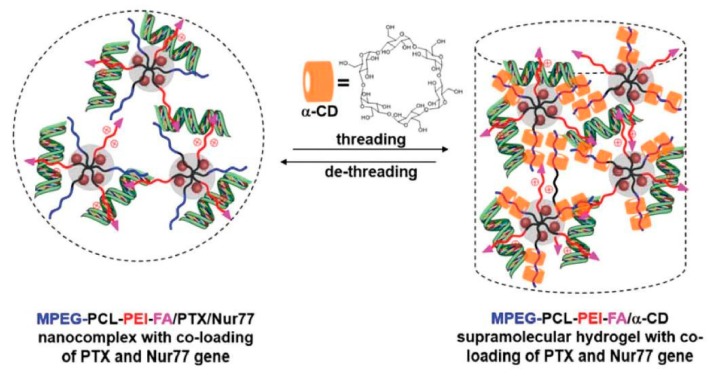
Illustration of the formation of the MPEG-PCL-PEI-FA/α-CD supramolecular hydrogel with co-loading of PTX and the Nur77 gene, which could also be reversed by the de-threading of α-CD in a biological environment, leading to the sustained released of the drug and gene. Adapted with permission from *Macromol. Rapid Commun*. **2019**, *40*, 1800117 [170]. Copyright (2018) WILEY-VCH Verlag GmbH & Co. KGaA, Weinheim.

Recently, the use of hybrid supramolecular polypseudorotaxane hydrogels with inorganic nanoparticles was also demonstrated. For example, Ren et al. [165] prepared pegylated magnetic (Fe_3_O_4_) and gold nanoparticles in the presence of poly(poly(ethylene glycol) methyl ether acrylate)-grafted poly(acrylic acid) or poly(2-(dimethylamino)ethyl methacrylate) copolymers. The pegylated nanoparticles were used for supramolecular hydrogel preparation by mixing them with α-CD. Magnetic and gold nanoparticle hybrid PPR hydrogels both showed temperature- and pH-responsiveness, thixotropic, and self-healing properties, as well as a sustainable nanoparticle release profile from the hydrogel matrix that was caused by water erosion. An external magnetic field manipulated the released magnetic nanoparticles to reach a specific location, which might be crucial for their use in the target delivery system. A similar PPR hydrogel that was hybridized with silver nanoparticles showed excellent antibacterial properties against various bacteria while exhibiting low cytotoxicity [166]. Supramolecular hybrid hydrogels were also used for drug delivery. Shi et al. [167] developed a simple biocompatible hydrogel for doxorubicin delivery by inclusion complexation between PEG-SH, gold nanoparticles, and α-CD. The introduction of gold nanoparticles resulted in a hybrid PPR hydrogel with improved mechanical properties and shear-thinning behavior, which is crucial for an injectable drug delivery system. Liu et al. [168] used a similar concept, designing and preparing poly(ethylene glycol)-luteolin–Fe_3_O_4_ conjugate micelles/α-CD PPR hydrogels for anticancer 5-fluorouracil drug delivery. The introduction of Fe_3_O_4_ particles into the hydrogel reduced the gelation time and improved the drug release profile. The polypseudorotaxane hydrogels were also explored as a controlled delivery system for gene transfer vectors. Wu et al. [169] developed a supramolecular hydrogel for therapeutic gene delivery. An injectable system was prepared from α-CD and poly(ethylene glycol)-b-poly(ε-caprolactone)-b-poly(ethylene imine) copolymer micelles with the ability to form polyplexes. Hydrogels possess a good controllable release effect of Bcl-2 conversion genes (Nur77). The encapsulation, followed by the controlled release of the antiapoptotic Bcl-2 protein in the systems, resulted in the effective inhibition of tumor growth after seven days in vivo when injected into a solid tumor. The same group further extended a similar concept. The hydrogel was formed by α-CD and poly(ethylene glycol)-b-poly(ε-caprolactone)-b-poly(ethylene imine) conjugated with folic acid as a vector group for tumors. A nanocomplex was formed by micelles that first condensed with DNA to form polyplexes, while paclitaxel was encapsulated in the hydrophobic poly(ε-caprolactone) core; subsequently, a supramolecular polypseudorotaxane hydrogel was formed by interactions with α-CD (Figure 9). The obtained supramolecular hydrogel was used for the synergistic co-administration of an anticancer drug (paclitaxel) and therapeutic gene (Nur77) as an efficient cancer treatment. Extensive in vivo studies showed that peritumoral injection of this hydrogel significantly hindered the growth of the tumor due to the sustained release of therapeutics and targeted tumor delivery by a folic acid vector [170].

Li et al. [171] prepared a hydrogel that was based on poly(ethylene glycol)-b-poly-(ε-caprolactone)-b-poly[2-(dimethylamino)ethyl methacrylate], which was used for gene delivery by condensing pDNA to make polyplexes and subsequently make a PPR hydrogel with α-CD. In vitro studies showed that the pDNA was released in the form of polyplex nanoparticles in a sustained manner for up to six days. The bioactivity of the released pDNA polyplexes was constant during the whole release experiment. Similarly, Zhang et al. [172] prepared PPR hydrogels for pDNA delivery from Pluronic F-68 (PEO-PPO-PEO) and poly(l-lysine) block copolymer. The cationic poly(l-lysine) block was able to condense pDNA to form polyplexes, with PEG chains on the micelle surface. With α-CD, it formed a hydrogel, and pDNA complexes were released from the hydrogel matrix in a controlled and sustained manner. Biocompatible heparin-conjugated Pluronic F-127 micelles/α-CD also prepared supramolecular hydrogel for the codelivery of an anticancer drug (camptothecin) and growth factor (granulocyte colony-stimulating factor) [173].

Stimuli responsiveness has also been shown in these supramolecular hydrogel systems. The inclusion of “intelligent” stimuli-sensitive moieties into these PPR systems allows for the synthesis of advanced materials that can respond in a controlled way upon application of certain triggers. For instance, Shuai et al. [174] prepared a pH-induced reversible gel–sol phase transition of the supramolecular hydrogel while using a poly(ethylene glycol)-b-poly(l-lysine) diblock copolymer to obtain micelles, which were used to create the PPR hydrogel with α-CD. The pH-inducible gelation of the hydrogel in water was completely reversible upon pH changes. The synergetic effect of selective complexation between PEG blocks and α-CD and the pH-inducible hydrophobic interaction between poly(l-lysine) blocks at pH 10 were the driving forces for the formation of the hydrogel. However, pH sensitivity can be used not only to force the gel–sol phase transition, but also as a trigger for anticancer drug release, since it is known that cancer tissues have a mildly acidic environment [175]. This is the reason that pH-responsive supramolecular hydrogels are broadly studied to develop novel systems for local anticancer drug delivery. Ni et al. [176] developed a pH-triggered biodegradable polypseudorotaxane hydrogel based on a star-block copolymer (mPEG-acetal-PCL-acetal-)_3_ by taking advantage of the acidic pH in tumors. The in vitro drug release showed that the anticancer drug (doxorubicin) was released from the drug-loaded hydrogel matrix in a controlled and pH-dependent manner. The same authors prepared an injectable supramolecular hydrogel that was formed by the interaction between α-CD and a pegylated doxorubicin prodrug linked with an acid-cleavable hydrazone group. The hydrogel exhibited lower cytotoxicity than the free doxorubicin and it degraded in the acidic environment of tumor cells, thereby releasing free doxorubicin [177]. The hydrazone linkage is commonly used in pH-responsive drug delivery systems. At pH > 7, the hydrazone bond is relatively stable and hydrolysis occurs very slowly, while, at pH 5–6, the rate of hydrolysis significantly increases [178]. Taking advantage of this property, Ji et al. [179] reported a pseudopolyrotaxane prodrug for enhanced tumor-targeted doxorubicin delivery. DOX was conjugated with α-CD via a hydrazone linkage. This approach allowed for a significant increase in the drug content in the hydrogel. The resulting hydrogel from the micelles of poly(ethylene glycol)-b-poly(2-methacryloyloxyethyl phosphorylcholine) and α-CD-hyd-DOX could respond to the endosomal pH, enabling controlled drug release in a mildly acidic environment. Recently, Jin et al. [180] prepared a pH- and oxidation-responsive hydrogel for dual controlled drug delivery. A polypseudorotaxane hydrogel that was based on pegylated poly(ether-urethane) with diselenide bond nanoparticles and α-CD were used to encapsulate the hydrophobic drug indomethacin into micelles and load hydrophilic rhodamine B into the hydrogel matrix. pH- and/or oxidation-triggered degradation of the supramolecular hydrogel controlled the release kinetics of the dual drugs. Liu et al. prepared a similar system [181]. The biocompatible and biodegradable hydrogel was synthesized by conjugating DOX via an acid-labile Schiff base linkage to the pegylated oxidized alginate, crosslinked with bioreducible disulfide bonds. Doxorubicin release from the loaded glycol chitosan–Pluronic F127 micelles was tested in vivo and then compared with conventional free DOX administration [182]. These tests revealed that peritumoral injection of the DOX-loaded PPR hydrogels significantly increased doxorubicin accumulation in tumor tissue and, at the same time, pH sensitivity prevented DOX accumulation in normal tissues. At the same administered drug dose, peritumoral injection of the hydrogel presented significantly better tumor therapeutic efficiency than intravenous injection of the drug-loaded micelles or free drug. These studies showed the superiority of the pH-responsive PPR hydrogels to classic anticancer drug administration [182].

Additionally, regarding various pH-responsive polypseudorotaxanes, thermosensitive hydrogels have also been prepared. Thermosensitivity was used to induce a reversible gel–sol phase transition at a particular temperature or to trigger drug release. As mentioned previously, tumoral temperature is higher than that of normal tissues [99]. Dai et al. [183] prepared a thermosensitive hydrogel for drug delivery based on binary drug-loaded micelles using eight-arm polyethylene glycol–betulinic acid/hydroxycamptothecin conjugates by taking advantage of the higher temperature in tumors. The thermosensitive hydrogel showed reversible gel–sol transition properties that were related to the structure at a particular temperature. Additionally, varying the length of the PEG chains and the concentration of cyclodextrin and nanoparticles controlled the gel–sol phase transition. In vivo studies showed that this hydrogel was more effective than free drugs and drug-loaded micelles. This same concept was extended while using micelles that were prepared from carboxymethylcellulose for betulinic acid/hydroxycamptothecine system delivery [184]. Similarly, Shi et al. [185] prepared a tunable temperature-responsive polypseudorotaxane hydrogel based on pegylated camptothecin, an amphiphilic anticancer prodrug. Moreover, the formed hydrogel was loaded with the hydrophilic drug 5-fluorouracil. Coadministration of these drugs from the hydrogel matrix significantly enhanced anticancer activity.

Light is also a useful trigger, because it can be precisely adjusted through its intrinsic properties, such as wavelength, intensity, or exposition time [186]. Recently, Shen et al. [187] designed and prepared an injectable, stimuli-responsive supramolecular hydrogel for the NIR-triggered delivery of cisplatin and repeatable chemo-photothermal combination therapy. Poly(N-phenylglycine)-poly(ethylene glycol) and α-CD, with the poly(N-phenylglycine) block serving as the NIR-absorbing moiety, comprised this PPR hydrogel. The NIR laser light triggers the gel–sol phase transition, affording subsequent cisplatin release, but it also mediates the photothermal ablation of cancer cells. This mode of treatment results in enhanced antitumor activity and reduced off-target toxicity. Cheng et al. [188] prepared a similar NIR-responsive supramolecular hydrogel that consisted of α-CD and a PEG-modified dendrimer encapsulated by platinum nanoparticles. Upon NIR irradiation, this hydrogel underwent degradation, with the subsequent release of the entrapped anticancer therapeutic (bortezomib) in an on-demand and dose-tunable manner. The conjugation of a photosensitive moiety with α-CD is another approach to obtaining photoresponsive PPR. Ji et al. [189] synthesized a pseudopolyrotaxane hydrogel that was prepared from chlorin e6 conjugated with α-CD via a glutathione-cleavable disulfide linker and fully biocompatible poly(ethylene glycol)-b-poly(2-methacryl-oyloxyethyl phosphorylcholine). Extended in vivo studies revealed that this system was stable and non-phototoxic, but it exhibited strong photodynamic theranostics in tumors. This pseudopolyrotaxane significantly enhanced the accumulation of chlorin e6 moieties in tumors, prolonged their tumor retention time, and improved their therapeutic effect when compared with free chlorin e6. Recently, Lv et al. [190] prepared a visible dual-fluorescent PPR hydrogel that was fabricated from indocyanine green-α-CD conjugate/porphyrin-PEG. It was used to track the disassembly of this hydrogel by fluorescence imaging. The hydrogel disintegration that is caused by NIR-laser irradiation is based on the photothermal effect of indocyanine green dye. The fluorescent signals of indocyanine green dye and porphyrin accurately tracked the in vivo hydrogel disintegration studies of modified α-CD and PEG, respectively, which enabled the fluorescence imaging tracking of each component derived from supramolecular hydrogel disintegration. This study provided an important basis for understanding the in vivo disintegration process of PPR hydrogels that were built from α-CD/PEG and will help to design injectable polypseudorotaxane hydrogels for the controlled release of biologically active substances.

## 4. Conclusions

**Figure 10:**
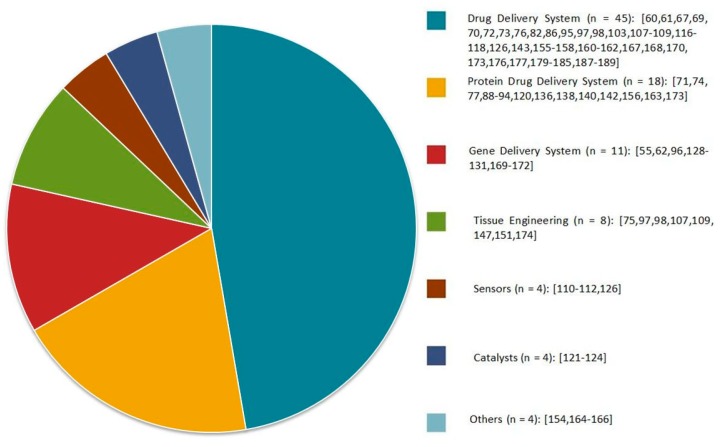
Applications of α-CD-based polypseudorotaxane hydrogels presented in the scientific literature in recent years. Citations concerning specific applications have been included.

During the last decade, there has been massive progress in the development of α-cyclodextrin-based polypseudorotaxane hydrogels because of their unique architecture and tunable rheological and physicochemical properties. The variety of “host” polymeric architectures, combined with stimuli-responsiveness and biocompatible α-CD, makes supramolecular polypseudorotaxane hydrogels ideal candidates for biomedical applications. Moreover, some efforts have been focused on the development of such hydrogels in various scientific fields for uses, such as catalysts, sensors, dye absorption, and diagnostics (Figure 10). In this review, α-CD-based PPR hydrogels are mostly discussed in the context of sustainable and target-oriented drug delivery systems. The successful implementation of such hydrogels as a strategy in the battle against cancer remains a great challenge, even though the current results are extremely promising. In future studies, smarter PPR hydrogel designs are expected for a superior balance between the clinical performance, practicality, and safety of these systems. It is expected that α-CD-based polypseudorotaxane hydrogels will play a significant role as drug carriers in the biomedical field.
